# More than a sum of parts: robust face recognition by integrating variation

**DOI:** 10.1098/rsos.172381

**Published:** 2018-05-30

**Authors:** Nadia Menon, Richard I. Kemp, David White

**Affiliations:** School of Psychology, UNSW, Kensington, Sydney 2052, Australia

**Keywords:** face recognition, identification, familiarity, memory integration

## Abstract

Familiarity incrementally improves our ability to identify faces. It has been hypothesized that this improvement reflects the refinement of memory representations which incorporate variation in appearance across encounters. Although it is established that exposure to variation improves face identification accuracy, it is not clear how variation is assimilated into internal face representations. To address this, we used a novel approach to isolate the effect of *integrating* separate exposures into a single-identity representation. Participants (*n* = 113) were exposed to either a single video clip or a pair of video clips of target identities. Pairs of video clips were presented as either a single identity (associated with a single name, e.g. Betty-Sue) or dual identities (associated with two names, e.g. Betty and Sue). Results show that participants exposed to pairs of video clips showed better matching performance compared with participants trained with a single clip. More importantly, identification accuracy was higher for faces presented as single identities compared to faces presented as dual identities. This provides the first direct evidence that the integration of information across separate exposures benefits face matching, thereby establishing a mechanism that may explain people's impressive ability to recognize familiar faces.

## 1. Introduction

We are adept at recognizing the faces of people we know, but this expertise does not extend to the faces of people we are not familiar with. Instead, unfamiliar face recognition is greatly impaired under conditions in which familiar face recognition remains robust. The gap between familiar and unfamiliar face recognition performance was demonstrated in a study by Bruce [1], who asked participants to study a series of face photographs, and then to recognize these faces in a subsequent test phase. In one condition, viewing angle and facial expression were changed between the study and test images. For familiar faces, these changes did not affect recognition accuracy, while for unfamiliar faces they resulted in a large drop in accuracy.

In another study [2], participants matched the identities in face photographs to poor-quality CCTV images. Participants who were personally familiar with the people in these images performed at around 90% accuracy, while participants who were not familiar with them were significantly less accurate. Importantly, the set of images being matched were the same for both groups of participants, and hence the differences between their performances cannot be attributed to differences in stimuli or task. Apparently, there is something about being familiar with a face that transforms the nature of face identification, and this transformation results in significant benefits to performance on identification tasks.

Several other studies have had similar outcomes, reporting marked increases in identification accuracy as a result of familiarity with a face [3,4]. Brief, laboratory-based familiarization procedures, which involve exposing participants to videos or photographs of previously unfamiliar faces, also improve subsequent identification performance [5–7]. Indeed, face matching performance improves incrementally with increased familiarity, leading to the suggestion that a viewer's performance on matching tasks can provide an index of their familiarity with a face [8–10].

The content of the memory representations that produce this improvement remains unclear. However, it is clear that such representations must tolerate significant variation in appearance, as the face of a single individual can look considerably different across encounters. Burton *et al*. [11] describe the sources of this *within-face* variability as reflecting changes in: (i) person-related factors, including changes in hairstyle, facial expression or changes related to age and (ii) image-related factors, such as camera angle, camera-to-subject distance or lighting conditions. A recent study has demonstrated that components of this variation, for example the nature of changes in expression, make-up or facial hair, carry identity information that is specific to individual identities [12]. This latter finding suggests that variation in appearance itself may be informative of facial identity, leading to the suggestion that person-specific variation may be encoded in memory representations of familiar faces.

Supporting this suggestion, when faces are unfamiliar, variation due to changes in appearance is often mistakenly interpreted as reflecting changes in identity. For example, when shown an array consisting of several photographs of multiple unfamiliar identities, participants consistently overestimate the number of identities present in the array [13–15]. This tendency is reduced by previous exposure to the faces, suggesting that familiarity engenders tolerance to variation across encounters with a face [13].

Further, face learning appears to be driven by exposure to variation. Studies have shown that experience of variation across instances of a face improves face identification. White *et al*. [16] found that if more than one image was provided of an unfamiliar ‘target' face, participants were better able to decide if a simultaneously presented ‘probe' photo was the same person as the target. Other studies have demonstrated that participants were better at finding a target face in a gallery of photos when given multiple photos of the target [17,18], and also show improved recognition memory performance as a function of the number of different photographs encountered during learning [14]. Recently, Menon *et al*. [19] demonstrated that learning an unfamiliar face from a highly variable set of photographs, rather than a less variable set, improved the ability to subsequently identify that face (see also [20]).

Critically, however, identification improvements resulting from exposure to a larger range of face images may be due to the increased probability of obtaining a single image which is particularly useful for identification (e.g. showing similar pose or expression to the probe photograph). According to this explanation, participants could represent each target instance separately, and only rely on information from this single ‘best' image when making a decision, while disregarding the other target images. Therefore, previous studies do not confirm that experience of variation *per se* is driving face learning. This conclusion depends on demonstrating that the advantage associated with experiencing a larger set of images of a target face is driven by integrating the variance *across* images, and not simply by the increased likelihood of obtaining an informative image.

Despite theoretical accounts emphasizing the importance of representing within-face variance [12,21,22], very little work explicitly investigates whether information is aggregated across multiple instances of a face. In the current experiment, we isolate the benefit of experiencing variation across separate face instances, over and above any benefit inferred from a particular instance, enabling us to determine whether experiencing variation is itself critical in the process of familiarization.

The current study explores the premise that multiple instances of a face contribute to a single face representation if they are thought to be a single person, and are represented separately if they are thought to be different people [23]. We modelled the process of familiarization by exposing participants to pairs of video clips that incorporate rigid (e.g. three-dimensional rotation) and non-rigid (e.g. changes in expression or speech) facial motion. Given the potential importance of variance in the process of familiarization, the video clips used in this study were not taken under controlled experimental conditions, but instead incorporate natural sources of variability in facial appearance, to better approximate our encounters with faces in daily life (as suggested by Burton *et al*. [11]). We then measured the benefit of this familiarity to face matching performance, which provides an index of face familiarity [8,10].

Critically, we also manipulated identity attributions to the exposures, by presenting pairs of videos of the same face either as a single identity (associated with a single name; e.g. Betty-Sue), or as two identities (associated with different names; e.g. Betty and Sue). If face representations integrate variation across multiple encounters, as has been suggested by recent theoretical accounts [11,12], then representations encoded as a single identity should confer more robust familiarity in comparison to representations encoded as dual identities, and should improve matching performance. In previous work, we have found that this encoding manipulation affects performance when matching static images [23], and we extend this investigation here using video clips.

## 2. Method

### 2.1. Participants

One hundred and thirteen first-year psychology students (76 female, mean age = 18.86 years) completed the experiment for course credit.

### 2.2. Stimuli

Thirty Europe-based minor celebrities were selected as target identities. These identities had been verified in previous experiments as being unfamiliar to Australian participants. For each target we collected two video clips of their face, taken from separate television appearances, and two ‘probe' photographs. Video clips were chosen by using the target's name as a search term in YouTube, and extracting a 2.5 s segment of each clip. Suitable segments were those in which the target was the only person visible, the face was unobscured, and engaged in both rigid and non-rigid motion. All other factors were free to vary (e.g. age, hairstyle, lighting angle, camera-to-subject distance). Video clips were cropped to a 2 : 3 aspect ratio, resized to 724 × 484 pixels and saved without audio.

For each target we also downloaded one still image of their face via Google Image search, in addition to one photo of a similar-looking ‘foil' identity (selected by the experimenter from our face database or Google Image). Photographs were free to vary on the same parameters as video clips, but we ensured that the face was pictured facing straight at the camera. All images were then cropped to a 2 : 3 aspect ratio, and scaled to 200 × 300 pixels. Target photographs appeared as probe images on match trials, and photos of foil identities were probe images on mismatch trials.

We assigned a double-barrelled name (e.g. Betty-Sue) to each stimulus identity. In the dual identity (2ID) condition, the names were associated separately with each target clip (i.e. one clip was ‘Betty' and the other ‘Sue’). In the single-clip (1CLIP) and single-identity (1ID) conditions, the clips were always associated with the double-barrelled name. Stimulus identities were randomly allocated to three groups. For each participant, each stimulus group appeared in one of the three experimental conditions; this was counterbalanced across participants.

### 2.3. Procedure

On each trial, participants initially learnt to associate names with target faces presented in video clips, before deciding if a probe photograph matched the people shown in the videos. Clips, names and photos were all presented centrally on a computer monitor. In an initial training phase, a target name was presented for 1 s. In 1CLIP and 1ID conditions, this was the full double-barrelled name (Betty-Sue) and in the 2ID condition this was a single-barrelled name (either Betty or Sue). The name was then replaced by the first video clip of that target and shown for 2.5 s. This was followed by a second name and video pairing. In the 1CLIP condition, participants were presented with the same name and video clip as before. In the 1ID and 2ID conditions, a second video clip was presented, but in the 1ID condition it was preceded by the same name as on the first presentation (e.g. Betty-Sue) and in the 2ID condition it was preceded by the alternative single-barrelled name (e.g. if Betty was seen previously, then Sue).

**Figure 1.**
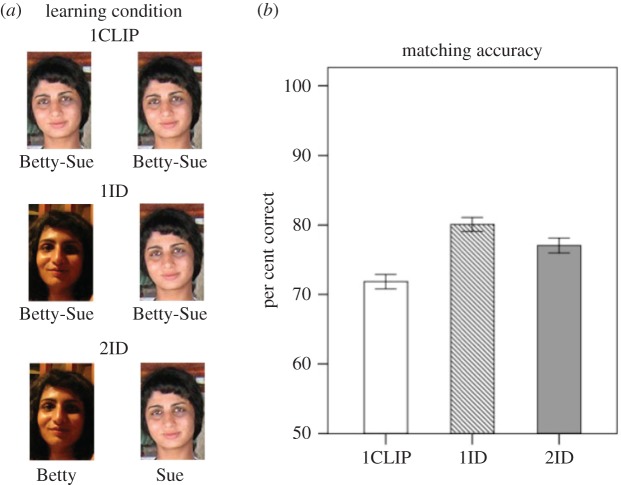
Illustration of the video-based face-learning procedure (*a*) and mean overall accuracy in each experimental condition (*b*). Error bars represent 1 s.e.m.

Thus, the same clips appeared in the 1ID and 2ID conditions; however, in the 2ID condition they were (misleadingly) presented as two different people (figure 1). The order of clip presentation in the 1ID and 2ID conditions was counterbalanced across participants. Immediately after the second clip was presented, the naming phase began. In the naming phase, the first clip was shown again, followed by a text input box, into which participants entered the name associated with that clip. They clicked a ‘continue' button when done. This sequence was then repeated for the second clip.

Immediately following this naming phase, the names and clips were presented in the same sequence as they had been presented in the initial training phase. The probe image was then presented and remained on screen until a response was made. In the 1CLIP and 1ID condition, participants decided whether the probe photo was the same identity as the target shown in the previous clips (i.e. *Is this Betty-Sue?*). In the 2ID condition, they decided if the probe matched *either* target (i.e. *Is this either Betty or Sue?*). Therefore, in all three conditions, participants were only required to make a ‘yes' or ‘no' response. Matching decisions were self-paced, with accuracy emphasized.

There were 60 trials in total, separated into 20 trial blocks by condition (in each condition participants would complete one match and one mismatch trial for each of 10 identities). The order of trials on each block was fully randomized. Block order was also randomized for each participant. After the experiment, we checked awareness of the identity manipulation, by asking participants if they had noticed anything surprising or unusual during the experiment. Given its importance to the study, an *a priori* decision was made to exclude participants who indicated they did not believe the 2ID manipulation. All participants were then fully debriefed. The experiment took approximately 1 h to complete.

## 3. Results

Five participants were excluded prior to analysis based on awareness of the identity manipulation, resulting in a sample of 108 participants (71 female, mean age 18.87 years).

Overall accuracy for the three conditions is shown in figure 1. We analysed overall accuracy in a one-way ANOVA with condition (1CLIP, 1ID, 2ID) as a within-subject factor. The main effect of condition was significant, *F*_2,214_ = 26.05, MSE = 72.12, *p* < 0.001, $\eta_{p}^{2}=0.196.$ Planned comparisons were conducted between each of the experimental conditions to confirm the multiple clip benefit (1ID/2ID versus 1CLIP), and to test for an effect of identity attribution (1ID versus 2ID).

First, we tested whether the benefit of multiple target instances shown in previous studies (e.g. [16]) was also replicated when using video clips rather than still photographs. It is possible that a single video clip would provide sufficient information about the possible variation in appearance of the target face, in which case the provision of a second clip would afford no further benefit to performance. However, matching accuracy in both 1ID (*F*_1,107_ = 51.39, MSE = 143.1, *p* < 0.001, $\eta_{p}^{2}=0.324$) and 2ID conditions (*F*_1, 107_ = 19.61, MSE = 148.1, *p* < 0.001, $\eta_{p}^{2}=0.155$) was higher than the 1CLIP condition, confirming the benefit of presenting multiple clips. More importantly for the aims of this experiment, the benefit was modulated by identity attributions: accuracy was greater in the 1ID compared to the 2ID condition, *F*_1,107_ = 7.174, MSE = 141.51, *p* = 0.009, $\eta_{p}^{2}=\text{0}.063$.

We also analysed accuracy separately for match and mismatch trials (table 1). For match trials, the main effect of condition was significant, *F*_2,214_ = 27.02, MSE* *= 215.4, *p *< 0.001, $\eta_{p}^{2}=0.202$. Planned comparisons for match trial performance mirrored the pattern evident in overall accuracy, whereby participants were more accurate in the 1ID condition compared to the 1CLIP condition (*F*_1,107_ = 50.88, MSE* *= 457.5, *p* < 0.001, $\eta_{p}^{2}=0.322$) as well as in the 2ID condition compared to the 1CLIP condition (*F*_1,107_ = 14.75, MSE* *= 422.2, *p *< 0.001, $\eta_{p}^{2}=\text{0}.121$). Critically, accuracy was higher in the 1ID than the 2ID condition (*F*_1,107_ = 13.14, MSE* *= 412.9, *p *< 0.001, $\eta_{p}^{2}=\text{0}.109$), showing that identity attributions affected performance on match trials.

**Table 1.** Mean (s.e.m.) accuracy (%) for match and mismatch trials in each condition.

	1CLIP	1ID	2ID
match	57.4 (*2.14*)	72.1 (*1.99*)	65.0 (*2.09*)
mismatch	86.3 (*1.17*)	88.1 (*1.23*)	89.1 (*1.24*)

For mismatch trials, the main effect of condition was not significant, *F*_2,214_ = 2.332, MSE* *= 92.21, *p *= 0.100, $\eta_{p}^{2}=\text{0}.021$. Planned comparisons revealed that mismatch accuracy was greater in the 2ID compared to the 1CLIP condition (*F*_1,107_ = 5.515, MSE* *= 151.1, *p* = 0.021, $\eta_{p}^{2}=0.049$), but not in the 1ID compared to the 1CLIP condition (*F*_1,107_ = 1.697, MSE = 211.1, *p* = 0.196, $\eta_{p}^{2}=0.016$). Mismatch accuracy was not affected by identity attributions, with no differences evident between the 1ID and 2ID conditions, *F*_1,107_ = 0.517, MSE* *= 191.1, *p* = 0.474, $\eta_{p}^{2}\;=0.005$.

## 4. Discussion

These results clearly demonstrate that information is aggregated across separate encounters with a face, improving the ability to subsequently identify that face. Firstly, overall identification accuracy was superior after exposure to pairs of video clips compared to a single clip, which is consistent with studies using static imagery [16,23]. Additionally, overall accuracy was higher when naming information encouraged the belief that these faces were of one person, despite the fact that the clips and images presented to participants were identical across both conditions. Therefore, it appears that integrating exposures into a unitary representation conferred an additional benefit, compared to encoding precisely the same information, but as two separate identities.

The results of this experiment provide direct evidence that abstractive identity-level representations combine variation across multiple exposures to support face matching. Previous studies have shown that exposure to variation improves accuracy in face identification tasks [16–18], with cumulative benefits observed with increasing exposure [14]. However, these earlier studies did not establish that the improvements depended on integrating information across multiple encounters with the target face. Here we have demonstrated that familiarity-based improvements in face identification confer benefits above and beyond the individual exposures contributing to this familiarity.

The benefit of presenting the two clips as a single rather than dual identity was evident on match trials, with accuracy on mismatch trials unaffected by identity attributions. In other words, when the clips were presented as one target identity, participants were specifically more likely to correctly decide that the probe image was the same person as the target. This pattern suggests that the memory representations generated in the one-identity condition were more tolerant of within-face variance in appearance, between the target clips and the probe image. This interpretation is consistent with the conclusion that participants in this condition integrated the variance across the clips into their representation of the target. It is also consistent with previous research demonstrating that increased familiarity with a face engenders tolerance of variance across images of that face [13].

Recent work has shown that components of variation across different images of the same face encode idiosyncratic parameters that are specific to particular faces [12]. This mirrors work aimed at improving automatic face recognition systems, where gains in performance have been attained by building identity-specific subspaces that encode variation specific to an individual [24]. Our results show that integration of within-person variation also drives accuracy in human subjects, supporting theoretical accounts of face representations that model familiarity as a process of aggregating variation [11,12].

There are other possible reasons why performance might have improved when the target clips were presented as a single identity. For example, when participants knew that both clips would be of a single target, they could potentially complete the task by choosing to attend to only one of the clips. On the other hand, when the two clips were presented as two target identities, participants would have to attend to both clips as they thought the probe might be a match for *either* target. Therefore, the task may have been less demanding in the 1ID than the 2ID condition. However, our data do not support this interpretation. Regardless of the identity attributions made to the clips, accuracy improved when two target clips were presented compared to when only one target clip was available, suggesting that both target clips were attended to in both conditions.

It is worth considering why single-identity encoding improved accuracy in the present study, which employed video clips, but not in a previous study, which used static images [23]. There is evidence that motion benefits face learning [6,25,26] possibly over the contribution of multiple static images [27]. Motion may improve face identification because it facilitates the representation of three-dimensional facial structure [27], or the facial motions which are specific to a particular identity may themselves be incorporated into our representations of that face [26,28]. In either case, representations based on multiple exposures to a moving face should be richer and more informative than those based on two static images, and hence more likely to confer a clear advantage to matching.

An additional possibility is that motion itself is necessary for *face*, as opposed to *image*, representation. Thornton & Kourtzi [26] have proposed that, at least in working memory, our representations for dynamic and static faces are qualitatively different. Pike *et al*. [27] make the point that, by their nature, photographs encourage pictorial representation, while dynamic objects may force the extraction of ‘object-centred' representation, or identity-specific representations, in the case of faces. By this logic, a face would need to be viewed in motion, in order to engage the processes responsible for aggregated representation, as described by Bruce & Young [21]. In support of this, Wallis *et al*. [29] demonstrated that motion allows for temporal association of different views of an object (in this case a face), which allows these views to be categorized as the same identity. Outside the laboratory, the faces we encounter are generally moving. Therefore, experiencing the target identities in motion in this study might have allowed for the formation of a face representation that better approximated our representations for familiar faces, and so provided a benefit to matching performance. In future work, it will be important to examine the role of motion in the formation of integrative representations across multiple encounters with a face.

In summary, we have demonstrated that identification accuracy is improved with increased experience of a face. We designed a novel method to isolate the effect of integrating variation across multiple face instances, and found that familiarity-based improvement was enhanced by such integration. Notably, asymptotic performance was not observed in any condition and hence future research should explore the trajectory of face familiarity as it unfolds over wider ranges of experience. We hope that the procedure used here to manipulate identity attribution can be a useful technique for such research, and for the study of invariant representations in other object domains.

## Supplementary Material

DataSet

## Data accessibility

The dataset supporting this article has been uploaded as part of the electronic supplementary material.
